# Systematic evaluation of three different commercial software solutions for automatic segmentation for adaptive therapy in head-and-neck, prostate and pleural cancer

**DOI:** 10.1186/1748-717X-7-160

**Published:** 2012-09-18

**Authors:** Mariangela La Macchia, Francesco Fellin, Maurizio Amichetti, Marco Cianchetti, Stefano Gianolini, Vitali Paola, Antony J Lomax, Lamberto Widesott

**Affiliations:** 1 Agenzia Provinciale per la Protonterapia, Via F.lli Perini, 181, 38122, Trento, Italy; 2 Istituto del Radio "O. Alberti", Spedali Civili, Brescia, Italy; 3 Center for Proton Radiation Therapy, Paul Scherrer Institute, Villigen PSI, Switzerland; 4 Department of Physics, Swiss Institute of Technology (ETH), Zurich, Switzerland

**Keywords:** Automatic segmentation, Adaptive radiotherapy, Re-planning

## Abstract

**Purpose:**

To validate, in the context of adaptive radiotherapy, three commercial software solutions for atlas-based segmentation.

**Methods and materials:**

Fifteen patients, five for each group, with cancer of the Head&Neck, pleura, and prostate were enrolled in the study. In addition to the treatment planning CT (pCT) images, one replanning CT (rCT) image set was acquired for each patient during the RT course. Three experienced physicians outlined on the pCT and rCT all the volumes of interest (VOIs). We used three software solutions (VelocityAI 2.6.2 (V), MIM 5.1.1 (M) by MIMVista and ABAS 2.0 (A) by CMS-Elekta) to generate the automatic contouring on the repeated CT. All the VOIs obtained with automatic contouring (AC) were successively corrected manually. We recorded the time needed for: 1) ex novo ROIs definition on rCT; 2) generation of AC by the three software solutions; 3) manual correction of AC.

To compare the quality of the volumes obtained automatically by the software and manually corrected with those drawn from scratch on rCT, we used the following indexes: overlap coefficient (DICE), sensitivity, inclusiveness index, difference in volume, and displacement differences on three axes (x, y, z) from the isocenter.

**Results:**

The time saved by the three software solutions for all the sites, compared to the manual contouring from scratch, is statistically significant and similar for all the three software solutions. The time saved for each site are as follows: about an hour for Head&Neck, about 40 minutes for prostate, and about 20 minutes for mesothelioma. The best DICE similarity coefficient index was obtained with the manual correction for: A (contours for prostate), A and M (contours for H&N), and M (contours for mesothelioma).

**Conclusions:**

From a clinical point of view, the automated contouring workflow was shown to be significantly shorter than the manual contouring process, even though manual correction of the VOIs is always needed.

## Introduction

Anatomical variations occurring during irradiation, including tumor shrinkage and shape deformation, can be significant and can result in suboptimal treatment of patients, especially when highly conformal treatment techniques, such as intensity modulated photon or proton therapy, are used [1,2].

Repeat imaging and replanning, even with a single mid-treatment CT scan only, can significantly improve tumor coverage and organ sparing in patients who experienced clinically apparent changes in anatomy [3,4]. However, in clinical practice adaptive planning is limited to a small number of patients considering the need for multiple physician-drawn volumes. To spread out the practice of adaptive radiotherapy easing the onerous task of recontouring is required.

In this context, a fast, robust, and automatic region-of-interest (ROI) delineation method is needed and has led to a growing array of automatic contouring (AC) software [5-7]. These algorithms generally deform one set of contours from an initial CT to fit the anatomy of a second CT, and they can also redraw the ROI of interest for each CT from scratch.

The purpose of the present study was to compare three different commercial algorithms applying them to a number of clinical cases. The software solutions (SS) were evaluated quantitatively both in terms of speed and reliability.

## Methods and materials

All volumes of interest (VOIs) were outlined manually by three in field specialized oncologists (e.g. H&N was contoured by a H&N oncologist, etc.) on planning CT (pCT), that represented our atlas for AC on three SS replanning CT (rCT).

We used three commercial software solutions to generate the automatic contours, subsequently all these VOIs were manually corrected (ACMC) by the same experienced physicians. The VOIs on the rCT were also manually contoured from scratch, that represented our reference volumes (Vref). The times employed for AC, for ACMC (when needed), and for Vref contouring were recorded. To evaluate the quality of the contours, AC and ACMC were compared, by the use of several parameters, with those manually delineated on rCT (Vref).

### Patient data and manual contouring

**Figure 1:**
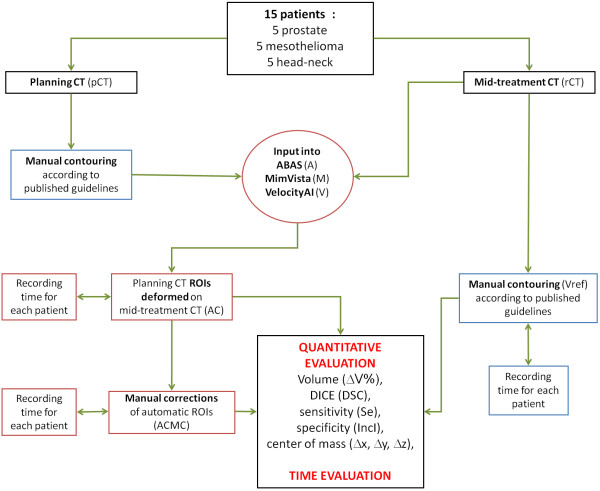
Flow chart showing input data and software evaluation strategy.

A conventional helical CT scanner was used for image acquisition. A total of 15 patients (five with locally-advanced head and neck (H&N) tumors, five with malignant pleural mesotheliomas (MPM) and five with high-risk prostate cancer (HRPCa)) were enrolled in the study. In addition to the treatment pCT images, a further set of CT images (rCT) was acquired for each patient during the RT course, usually in the middle of the treatment. For H&N, prostate and mesothelioma patients the images had a slice thickness of 3, 2.5, and 5 mm, respectively. A commercial treatment planning system (Focal 4D by Elekta, Sweden) was used for the manual contouring from scratch of ROIs (Figure 1).

All H&N patients (two oropharynx, one oral cavity, one nasopharynx, and one larynx) had pathologically confirmed Stage III-IV disease. The target volumes were determined according to the ICRU definition of GTV and CTV. For each patient, neck levels were delineated according to international consensus guidelines [8]. In addition, 16 organs at risk (OARs) were contoured (parotid, cochlea, esophagus, brainstem, spinal cord, mandible, thyroid, pharynx, masticatory spaces, larynx, oral cavity, temporal lobes, eyes, lenses, optic nerves and chiasm). Five MPM patients had been previously treated with extraperitoneal pleuro-pneumonectomy and received adjuvant thoracic irradiation. The CTV included the entire hemithorax and thoracotomy incision and site of chest drains. Two patients had a lesion on the right side and three on the left. Contoured normal tissues were: contralateral lung, heart, esophagus, liver, bowel, spinal cord, spleen, kidneys. Five HRPCa patients (PSA > 20 ng/mL; Gleason score 8–10 or c/pT3a/b) [9] had been previously treated, two with definitive radiotherapy and three with post-operative irradiation. The CTV encompassed the prostate and seminal vesicles (definitive irradiation) or prostatic bed (post-operative irradiation) and pelvic lymphnodes. The defined OARs were: rectum, bladder, femoral heads and bowel.

### Automatic contouring

Using three commercially available programs a) ABAS 2.0 (CMS-Elekta, Stockholm, Sweden) (A), b) MIM 5.1.1, (MIMVista corp, Cleveland, Ohio) (M), and c) VelocityAI 2.6.2 (Velocity Medical Systems, Atlanta, Georgia) (V), contours from the initial pCT were deformed to the replanning CT for each patient.

In ABAS an atlas patient consists of a CT scan with pre-defined ROIs, both target volumes and OARs. A detailed description of the method has been published by Han et al. [10]. Firstly, non-rigid registration is used to transform the CT scan of an atlas patient (pCT) to replanning CT scan. Specific models for e.g., H&N and prostate are available in the software, taking structure-specific information, like elasticity, into account. Then, using the obtained transformation, auto-contours are generated by mapping the atlas contours to the replanning CT scan.

Also in MIM, we decided to use a single-atlas segmentation approach: the pCT of the patient was inserted into the atlas and, subsequently, the algorithm extracts information from one CT to generate the automated contour of the rCT. In order to do this, initially rigid registration with rotations were applied, followed by deformable registration. The previously validated intensity-based free-form deformable registration algorithm utilizes regularization to minimize the likelihood of folds or tears in the deformation fields to fit one CT to another [11].

A single-patient-atlas segmentation approach was used also with VelocityAI. Between planning CT and replanning CT, we firstly applied a rigid registration with rotations and secondly, a deformable multi pass registration. Finally, we copied the contours from planning CT to replanning CT and this software applied automatically the deformation matrix to them. VelocityAI uses the basis-spline (B-spline) method [12] for deformable registrations.

### Time/speed evaluation

Focal 4D, ABAS and MIM were installed in a 3 GHz HP xw 8600 workstation running Windows with 8 GB RAM, whereas VelocityAI was installed in a 2.66 GHz HP xw 8600 workstation running Windows with 4 GB RAM. The time required for the ex novo ROIs definition on rCT, for the three software solutions to generate the AC, and finally, the time for manual correction of AC was calculated.

The time to manually define the volumes on rCT was calculated from the opening of the latest CT to the last ROI. The time needed by the software solutions to generate automatic contours was measured by when the CT was imported until the end of the entire generation process. The time needed to check the automatically-obtained volumes was defined from the time of loading the CT until the time needed for final volume correction (Figure 1). The usefulness of the automatic contours procedure was evaluated by comparing 1) the time needed from the software + manual corrections vs. manual contour from scratch or 2) just the manual correction time vs. manual contour from scratch (i.e. not considering the time needed by the computer for the generation of the deformed contours).

### Quantitative evaluation of automated and manually corrected contours

The performance of the automatic segmentation software was assessed by quantitatively comparing manual Vref contours with AC or ACMC contours in terms of volume, position and shape. A sensitivity and specificity study was also conducted. Manual segmentation was used as the reference segmentation.

As an initial measure of the similarity between the automatic and manual contours, the volume of every structure was calculated and the difference between the automatically generated volume (V_AA_, V_MA_, V_VA_, for ABAS, MIM and VElocityAI, respectively) and the manually generated volume, or reference volume, was calculated for each structure, as follows:

$$\Delta V\left(\%\right)=\frac{V_{\mathrm{auto}}-V_{\mathrm{ref}}}{V_{\mathrm{ref}}}\cdot 100$$

Also the difference between manually corrected automatic volume (V_AM_, V_MM_, V_VM_, for ABAS, MIM and VElocityAI respectively) and the Vref was calculated.

Since its introduction, DICE similarity coefficient (DSC) index [13] has been widely used in the evaluation of deformable image registration results. The DSC index is defined as

$$DSC=\frac{2\cdot \left(V_{\mathrm{ref}}\cap V_{\mathrm{auto}}\right)}{V_{\mathrm{ref}}+V_{\mathrm{auto}}}$$

Vref were compared to automatically contoured ROIs (or manually corrected after automatic generation ROIs). DSC values range from 0 to 1, and are identical to 1 if automatic and manual volumes were equal with a complete intersection.

For all the software solutions evaluated, the sensitivity index (Se) of contours was computed as:

$$Se=\frac{V_{\mathrm{ref}}\cap V_{\mathrm{auto}}}{V_{\mathrm{ref}}}$$

The sensitivity reflects the probability that the automatic contours (before or after the manual corrections) match the reference contour and some authors renamed it as the overlapping index (OI) [7].

We defined, as a surrogate of the specificity, the inclusiveness index (IncI):

$$IncI=\frac{V_{\mathrm{ref}}\cap V_{\mathrm{auto}}}{V_{\mathrm{auto}}}$$

The inclusiveness index reflects the inclusion of $V_{\mathrm{auto}}$ within $V_{\mathrm{ref}}$, i.e. the probability that a voxel of the $V_{\mathrm{auto}}$is really a voxel of the $V_{\mathrm{ref}}$.

To help the reader get an idea of some parameter trends, a modified Receiver Operating Characteristic (mROC) analysis was done by plotting the sensitivity vs. (1 _ IncI) for some delineated structure. The best possible result was expected to yield a point in the upper left corner or coordinate (0, 1) of the ROC space, representing 100% sensitivity (all voxels are true positive) and 100% of inclusion (surrogate of specificity, i.e. no false positive voxel is present).

As a general measure for the location of the structures, for each patient and for each structure (manually defined from scratch (i.e. reference structure), automatically generated and manually corrected after automatic generation) mass centre is calculated and the distance in the three coordinates was evaluated:

$$\Delta x=\left|x_{\mathrm{auto}}-x_{\mathrm{ref}}\right|;\Delta y=\left|y_{\mathrm{auto}}-y_{\mathrm{ref}}\right|;\Delta z=\left|z_{\mathrm{auto}}-z_{\mathrm{ref}}\right|$$

As reported in Figure 1, in order to evaluate these parameters in a systematic and consistent way, all DICOM images and structures were exported to VODCA4rt (MSS GmbH, Hagendorn, Switzerland) version 4.4.1. Therefore, we used the analysis tool box of VODCA for the automatic calculation of the variables described above.

A non-parametric Wilcoxon signed rank test was used to determine whether or not the observed differences were statistically significant. The Holm-Bonferroni correction was considered as well.

## Results

### Time/speed evaluation

Table 1 shows the average time needed for manual contouring, the software time for AC and correction time for the VOIs on rCT for each anatomical site. The differences in AC time between A, M and V are always statistically significant.

**Table 1:** Mean contouring duration, for the five patients, for each tumor considered

**Anatomical sites**	**Manual contouring time (SD)**	**Software**	**Automatic contouring time (SD)**	**Manual corrections time (SD)**	**Automatic+corrections**
PROSTATE	1.18.37 (0.09.23)	ABAS	0.14.51 (0.00.39)	0.32.42 (0.06.37) * °	* § =
		MIM	0.02.34 (0.00.12)	0.20.31 (0.05.12) * # +	* % =
		VELOCITY AI	0.08.23 (0.00.16)	0.28.35 (0.05.40) * °	* % §
HEAD & NECK	2.43.12 (0.48.21)	ABAS	0.10.58 (0.02.21)	1.09.15 (0.13.49) * ° +	* § =
		MIM	0.03.46 (0.00.35)	1.35.30 (0.17.11) * #	* %
		VELOCITY AI	0.06.21 (0.00.37)	1.52.30 (0.5.46) * #	* %
MESOTHELIOMA	1.21.02 (0.05.00)	ABAS	0.08.32 (0.00.17)	0.50.24 (0.03.35) * ° #	* =
		MIM	0.02.35 (0.00.17)	0.56.00 (0.02.21) * #	*
		VELOCITY AI	0.06.47 (0.00.22)	0.59.36 (0.09.04) * #	* %

- For manual corrections time:

"*" = vs. Manual contour from scratch: p<0.05.

"#" = vs. Manual correction from ABAS: p<0.05.

"°" = vs. Manual correction from MIM: p<0.05.

"+" = vs. Manual correction from VelocityAI: p<0.05.

- For automatic+corrections:

"*" = vs. Manual contour from scratch: p<0.05.

"%" = vs. ABAS+ Manual correction from ABAS: p<0.05.

"§" = vs. MIM + Manual correction from MIM: p<0.05.

"=" = vs. Velocity AI + Manual correction from VelocityAI: p<0.05.

After comparing the sum of the average duration of automatic + manual correction for each site and software with the total time needed to get the Vref, we can conclude the following: 1) for prostate patients, MIM was the software that obtained the most gain in time (55 min), while the average gain was 31 min and 41 min with ABAS and VelocityAI respectively; 2) for the H&N site, ABAS was the most time saving software with an average gain of 1 hour and 23 min; MIM can save 1 hour and 4 min, and VelocityAI 45 min; 3) for the mesothelioma cases, the average obtained gain was 22 min with both ABAS and MIM, and 15 min with Velocity. For all sites, the time gain for all three software solutions, compared to manual contouring from scratch, is statistically significant. In Table 1 we also reported the time needed for manual correction, aside from the time needed for the automatic contouring as the reader may be interested in the ‘physician time’ saved, independently from the time the software takes to generate the automatic contours.

### Contour evaluation

Regarding the DSC index for structures located in the pelvis, an important example is given by the rectum. Table 2 shows how similar this index is for all three software solutions, with regards to the AC (AA = 0.77, MA = 0.75, VA = 0.75). However, it is quite far from the value 1; this means that all three VOIs, which were obtained automatically, are not qualitatively close to the Vref. After manual correction of the automatic contours, the DSC improved for all the software solutions but the values of this index still remained ≤ 0.9. For this organ we found a ΔV from about +30% to −15% for AA and MA respectively that was reduced to about 5% after the manual correction. The Δz values for rectum (before and after manual correction) underlined the difficulty in contouring the cranio-caudal limits of this structure. The three software solutions tended to give us CTV1 and bowel volumes smaller than the Vref. VelocityAI had a lower sensitivity index, also after manual correction, compared to the other two softwares.

**Table 2:** Mean values and standard deviations of parameters that evaluate the contours generated by the three software, before and after the manual correction, for each organ of the prostate patients

	**AA**	**MA**	**VA**	**AM**	**MM**	**VM**	**p value**
**CTV1**							
DSC, (SD)	0.86 (0.08)	0.79 (0.05)	0.76 (0.14)	0.88 (0.09)	0.81 (0.04)	0.82 (0.02)	
Sensitivity, (SD)	0.82 (0.11)	0.72 (0.11)	0.70 (0.20)	0.85 (0.11)	0.76 (0.09)	0.82 (0.07)	
IncI, (SD)	0.92 (0.05)	0.89 (0.07)	0.85 (0.06)	0.91 (0.06)	0.88 (0.03)	0.84 (0.07)	*
ΔV (%), (SD)	−11.48 (7.83)	−17.83 (17.60)	−17.70 (23.61)	−7.73 (6.32)	−13.15 (12.11)	−1.30 (15.18)	°
Δx (mm), (SD)	0.45 (0.33)	0.35 (0.24)	0.38 (0.52)	0.35 (0.24)	0.55 (0.40)	0.85 (0.58)	
Δy (mm), (SD)	0.60 (0.64)	0.93 (0.56)	0.68 (0.56)	1.03 (1.01)	0.83 (0.50)	0.88 (0.90)	
Δz (mm), (SD)	1.25 (1.44)	3.18 (2.39)	1.88 (1.25)	1.28 (1.02)	2.23 (1.19)	2.20 (0.60)	
**CTV2**							
DSC, (SD)	0.77 (0.06)	0.86 (0.01)	0.83 (0.02)	0.87 (0.08)	0.87 (0.01)	0.84 (0.01)	* ° =
Sensitivity, (SD)	0.67 (0.12)	0.87 (0.02)	0.84 (0.03)	0.86 (0.09)	0.87 (0.03)	0.85 (0.03)	* ° =
IncI, (SD)	0.93 (0.06)	0.85 (0.02)	0.82 (0.04)	0.88 (0.08)	0.86 (0.03)	0.84 (0.02)	* #
ΔV (%), (SD)	−27.46 (17.74)	2.54 (4.98)	2.78 (8.47)	−1.44 (8.11)	1.88 (7.6)	2.12 (6.31)	* #
Δx (mm), (SD)	1.63 (1.37)	0.45 (0.41)	0.35 (0.47)	0.98 (1.13)	0.48 (0.41)	0.58 (0.22)	
Δy (mm), (SD)	3.54 (1.28)	2.58 (0.92)	2.55 (1.30)	2.23 (2.62)	2.85 (0.93)	2.68 (1.03)	
Δz (mm), (SD)	1.28 (1.79)	1.28 (1.79)	1.90 (1.62)	1.58 (1.90)	2.23 (1.85)	1.90 (1.63)	
**Rectum**							
DSC, (SD)	0.77 (0.07)	0.75 (0.09)	0.75 (0.04)	0.90 (0.07)	0.87 (0.05)	0.86 (0.05)	
Sensitivity, (SD)	0.88 (0.06)	0.70 (0.17)	0.72 (0.13)	0.92 (0.08)	0.89 (0.05)	0.87 (0.07)	* # ^
IncI , (SD)	0.69 (0.11)	0.84 (0.06)	0.79 (0.08)	0.88 (0.07)	0.86 (0.09)	0.86 (0.09)	
ΔV (%), (SD)	30.2 (24.30)	−15.38 (24.1)	−7.06 (23.65)	4.7 (10.10)	5.2 (13.88)	2.34 (16.64)	* °
Δx (mm), (SD)	2.00 (1.78)	2.14 (1.46)	3.36 (1.70)	1.30 (2.11)	0.720 (0.71)	1.92 (1.70)	
Δy (mm), (SD)	2.74 (1.81)	1.36 (0.85)	1.50 (1.71)	1.44 (1.15)	0.90 (0.30)	1.16 (0.76)	
Δz (mm), (SD)	6.54 (5.33)	6.02 (5.25)	6.04 (4.16)	3.78 (4.05)	6.8 (3.71)	7.28 (5.11)	^
**Bladder**							
DSC, (SD)	0.93 (0.03)	0.88 (0.07)	0.72 (0.15)	0.95 (0.04)	0.93 (0.02)	0.93 (0.02)	# °
Sensitivity, (SD)	0.93 (0.03)	0.88 (0.12)	0.67 (0.25)	0.96 (0.03)	0.96 (0.03)	0.93 (0.05)	=
IncI, (SD)	0.92 (0.06)	0.89 (0.04)	0.89 (0.13)	0.94 (0.04)	0.91 (0.03)	0.93 (0.05)	+
ΔV (%), (SD)	1.88 (8.29)	−1.70 (14.87)	−20.24 (43.21)	2.40 (3.90)	6.12 (5.89)	0.20 (8.79)	=
Δx (mm), (SD)	1.10 (0.16)	0.46 (0.46)	0.90 (0.67)	0.66 (0.53)	0.46 (0.46)	0.86 (1.01)	
Δy (mm), (SD)	1.32 (1.53)	3.42 (3.98)	3.44 (4.06)	0.52 (0.56)	0.72 (0.57)	0.76 (0.34)	
Δz (mm), (SD)	1.80 (0.66)	2.02 (1.12)	5.02 (2.92)	2.02 (2.28)	1.80 (1.19)	2.02 (1.43)	^
**Bowel**							
DSC, (SD)	0.83 (0.10)	0.85 (0.09)	0.88 (0.03)	0.89 (0.12)	0.92 (0.02)	0.87 (0.09)	=
Sensitivity, (SD)	0.79 (0.15)	0.82 (0.14)	0.88 (0.03)	0.88 (0.18)	0.94 (0.02)	0.84 (0.15)	=
IncI, (SD)	0.89 (0.08)	0.90 (0.04)	0.88 (0.05)	0.91 (0.05)	0.91 (0.03)	0.91 (0.01)	
ΔV (%), (SD)	−10.78 (17.74)	−9.12 (14.13)	−0.10 (6.50)	−3.38 (18.54)	2.36 (2.98)	−7.82 (15.27)	
Δx (mm), (SD)	3.44 (2.63)	2.58 (1.84)	3.32 (3.06)	2.16 (1.79)	2.98 (1.40)	3.46 (3.36)	
Δy (mm), (SD)	8.42 (3.68)	6.88 (3.90)	5.34 (3.72)	4.20 (5.56)	7.48 (3.82)	4.78 (5.87)	
Δz (mm), (SD)	9.78 (10.21)	9.78 (12.15)	6.28 (4.43)	6.02 (11.39)	0.26 (0.58)	5.00 (11.18)	
**Femoral head right**							
DSC, (SD)	0.94 (0.04)	0.94 (0.02)	0.92 (0.03)	0.94 (0.04)	0.94 (0.01)	0.93 (0.02)	# ° =
Sensitivity, (SD)	0.94 (0.05)	0.96 (0.02)	0.94 (0.03)	0.94 (0.05)	0.95 (0.02)	0.95 (0.03)	°
IncI , (SD)	0.94 (0.04)	0.93 (0.03)	0.90 (0.04)	0.95 (0.04)	0.93 (0.03)	0.91 (0.03)	* # ° + ^ =
ΔV (%), (SD)	0.12 (4.98)	2.92 (4.63)	4.82 (4.53)	−0.18 (4.92)	2.76 (4.98)	4.22 (5.08)	°
Δx (mm), (SD)	0.16 (0.36)	0.36 (0.21)	0.46 (0.49)	0.16 (0.36)	0.36 (0.21)	0.64 (0.31)	
Δy (mm), (SD)	0.18 (0.25)	0.36 (0.38)	0.74 (0.51)	0.26 (0.24)	0.36 (0.38)	0.92 (0.67)	
Δz (mm), (SD)	1.76 (1.11)	1.76 (1.70)	1.76 (2.09)	1.78 (1.43)	2.02 (0.66)	2.04 (1.11)	
**Femoral head left**							
DSC, (SD)	0.94 (0.04)	0.94 (0.03)	0.92 (0.02)	0.94 (0.04)	0.94 (0.02)	0.92 (0.03)	* # ^
Sensitivity, (SD)	0.95 (0.04)	0.96 (0.02)	0.94 (0.02)	0.95 (0.04)	0.96 (0.02)	0.95 (0.03)	° =
IncI , (SD)	0.93 (0.05)	0.92 (0.05)	0.90 (0.04)	0.94 (0.05)	0.92 (0.04)	0.90 (0.05)	# ^
ΔV (%), (SD)	2.30 (5.73)	4.92 (6.06)	5.42 (6.47)	1.24 (6.01)	4.94 (5.70)	4.94 (7.35)	* # + ^
Δx (mm), (SD)	0.62 (0.91)	0.68 (0.67)	0.66 (0.49)	0.62 (0.91)	0.68 (0.67)	0.76 (0.44)	
Δy (mm), (SD)	0.16 (0.36)	0.36 (0.38)	0.64 (0.31)	0.16 (0.36)	0.36 (0.38)	0.76 (0.51)	
Δz (mm), (SD)	3.28 (1.91)	2.26 (1.85)	2.02 (1.92)	2.02 (1.43)	1.52 (1.04)	2.30 (1.37)	

For automatic segmentation: *AA vs. MA: p<0.043, #AA vs. VA: p<0.043, °MA vs. VA: p<0.043.

For automatic segmentation+ manual correction: +AM vs. MM: p<0.043, ^AM vs. VM: p<0.043, =MM vs. VM: p<0.043.

*Abbreviations: CTV* = Clinical target volume; *SD* = Standard deviation, *DSC* = DICE similarity coefficient, *IncI* = inclusiveness index, *AA* = Abas Automatic, *MA* = MiM Automatic, *VA* = Velocity Automatic, *AM* = Abas Manual Correction, *MM* = MiM Manual Correction, *VM* = Velocity Manual Correction.

Regarding the H&N cases (Table 3), there were no statistically significant differences between the three software solutions for the CTV2, larynx, and superior part of the larynx. Generally, after manual correction, ABAS showed a higher inclusiveness index whereas MIM showed a higher sensitivity. From the ΔV parameter analysis, we found that ABAS tended to underestimate the volume of the VOI, while MIM and VelocityAI tended to overestimate it.

**Table 3:** Mean values and standard deviations of parameters that evaluate the contours generated by the three software, before and after the manual correction, for each organ of the head and neck

	**AA**	**MA**	**VA**	**AM**	**MM**	**VM**	**p value**
**CTV1**							
DSC, (SD)	0.68 (0.19)	0.67 (0.17)	0.66 (0.18)	0.72 (0.15)	0.76 (0.11)	0.71 (0.09)	#
Sensitivity, (SD)	0.73 (0.23)	0.76 (0.19)	0.74 (0.22)	0.79 (0.18)	0.85 (0.10)	0.81 (0.16)	
IncI, (SD)	0.64 (0.17)	0.61 (0.18)	0.60 (0.17)	0.67 (0.13)	0.69 (0.13)	0.65 (0.09)	* #
ΔV (%), (SD)	14.10 (25.99)	25.92 (21.18)	23.28 (28.44)	17.72 (22.19)	24.94 (26.01)	27.40 (32.49)	
Δx (mm), (SD)	2.62 (2.02)	2.92 (1.76)	2.44 (1.58)	2.12 (1.45)	2.70 (1.03)	2.88 (2.42)	
Δy (mm), (SD)	2.58 (2.70)	2.66 (2.72)	2.86 (2.37)	2.60 (2.47)	2.74 (2.47)	5.30 (3.32)	
Δz (mm), (SD)	5.40 (7.98)	5.70 (5.65)	5.10 (5.77)	5.40 (7.98)	3.60 (5.67)	4.80 (5.02)	
**CTV2**							
DSC, (SD)	0.84 (0.03)	0.83 (0.03)	0.81 (0.02)	0.85 (0.03)	0.84 (0.03)	0.82 (0.03)	
Sensitivity, (SD)	0.85 (0.04)	0.86 (0.05)	0.85 (0.05)	0.86 (0.04)	0.88 (0.04)	0.86 (0.04)	
IncI, (SD)	0.83 (0.06)	0.81 (0.06)	0.78 (0.05)	0.83 (0.06)	0.81 (0.06)	0.78 (0.06)	
ΔV (%), (SD)	3.50 (11.31)	7.40 (13.33)	10.00 (12.34)	4.45 (10.20)	8.38 (11.57)	10.68 (12.51)	
Δx (mm), (SD)	2.95 (2.30)	2.85 (1.85)	3.08 (1.81)	2.48 (2.56)	2.95 (1.80)	3.38 (1.37)	
Δy (mm), (SD)	2.38 (0.69)	3.73 (2.50)	2.53 (1.94)	2.23 (0.95)	3.60 (2.54)	2.90 (1.51)	
Δz (mm), (SD)	1.50 (1.23)	1.50 (2.12)	1.88 (2.25)	1.50 (1.23)	1.88 (1.44)	2.63 (1.44)	
**Larynx**							
DSC, (SD)	0.86 (0.04)	0.87 (0.01)	0.82 (0.04)	0.90 (0.03)	0.89 (0.02)	0.86 (0.05)	
Sensitivity, (SD)	0.87 (0.06)	0.88 (0.03)	0.85 (0.06)	0.92 (0.04)	0.93 (0.01)	0.91 (0.02)	
IncI, (SD)	0.86 (0.03)	0.85 (0.03)	0.80 (0.05)	0.87 (0.02)	0.85 (0.02)	0.82 (0.08)	
ΔV (%), (SD)	1.43 (5.92)	3.85 (6.75)	5.83 (9.59)	5.15 (3.37)	8.55 (1.93)	11.20 (11.32)	
Δx (mm), (SD)	0.65 (0.51)	0.38 (0.48)	0.88 (0.48)	0.90 (0.52)	0.53 (0.61)	0.50 (0.00)	
Δy (mm), (SD)	0.38 (0.25)	0.75 (0.65)	0.40 (0.80)	0.38 (0.25)	0.50 (0.58)	0.90 (0.52)	
Δz (mm), (SD)	5.63 (3.75)	9.75 (5.81)	3.75 (4.50)	0.75 (0.87)	1.50 (1.23)	0.75 (1.50)	
**Sup. Pharynx**							
DSC, (SD)	0.36 (0.22)	0.35 (0.12)	0.33 (0.13)	0.57 (0.05)	0.50 (0.14)	0.52 (0.11)	
Sensitivity, (SD)	0.49 (0.38)	0.51 (0.35)	0.47 (0.32)	0.74 (0.10)	0.80 (0.11)	0.76 (0.11)	
IncI, (SD)	0.42 (0.14)	0.42 (0.26)	0.40 (0.23)	0.49 (0.13)	0.40 (0.20)	0.42 (0.19)	
ΔV (%), (SD)	27.70 (90.39)	67.50 (125.10)	57.25 (112.28)	61.63 (48.64)	146.60 (134.73)	107.58 (96.01)	
Δx (mm), (SD)	1.18 (1.41)	1.43 (1.27)	1.73 (2.45)	0.98 (0.95)	0.75 (0.50)	1.03 (0.82)	
Δy (mm), (SD)	1.88 (1.38)	1.90 (1.04)	3.23 (3.78)	1.13 (0.90)	1.48 (1.63)	1.88 (1.74)	
Δz (mm), (SD)	6.00 (3.24)	6.75 (3.12)	4.88 (3.75)	3.00 (1.73)	4.50 (2.12)	3.38 (3.09)	
**Mid. Pharynx**							
DSC, (SD)	0.57 (0.10)	0.56 (0.13)	0.43 (0.19)	0.61 (0.09)	0.60 (0.12)	0.62 (0.12)	
Sensitivity, (SD)	0.52 (0.12)	0.57 (0.17)	0.44 (0.19)	0.59 (0.12)	0.68 (0.15)	0.64 (0.13)	°
IncI, (SD)	0.65 (0.13)	0.56 (0.12)	0.44 (0.21)	0.64 (0.10)	0.55 (0.13)	0.61 (0.11)	* #
ΔV (%), (SD)	−18.94 (18.43)	4.62 (27.73)	4.12 (37.64)	−7.48 (16.77)	27.38 (34.84)	4.66 (9.21)	* +
Δx (mm), (SD)	0.60 (0.82)	0.80 (0.45)	1.20 (1.11)	0.90 (0.82)	0.80 (0.45)	0.70 (0.57)	
Δy (mm), (SD)	3.32 2.64()	2.78 (1.08)	2.64 (2.256)	3.02 (2.78)	2.76 (2.32)	1.68 (1.29)	
Δz (mm), (SD)	4.20 (4.55)	3.90 (4.70)	4.80 (5.13)	4.20 (4.42)	5.40 (4.81)	5.10 (5.37)	
**Inf. Pharynx**							
DSC, (SD)	0.65 (0.07)	0.66 (0.05)	0.63 (0.07)	0.71 (0.04)	0.72 (0.03)	0.72 (0.04)	
Sensitivity, (SD)	0.59 (0.08)	0.65 (0.08)	0.60 (0.09)	0.67 (0.04)	0.72 (0.10)	0.73 (0.07)	*
IncI, (SD)	0.75 (0.11)	0.69 (0.07)	0.66 (0.09)	0.75 (0.10)	0.72 (0.05)	0.72 (0.09)	# ^
ΔV (%), (SD)	−20.04 (15.57)	−5.58 (17.12)	−7.46 (16.01)	−9.96 (14.02)	1.62 (19.64)	3.22 (18.60)	* #
Δx (mm), (SD)	1.12 (0.44)	0.72 (0.47)	0.90 (0.65)	0.60 (0.55)	0.50 (0.50)	0.70 (0.48)	
Δy (mm), (SD)	2.06 (2.28)	1.62 (1.63)	1.38 (1.43)	1.90 (2.12)	1.52 (0.61)	1.28 (2.07)	
Δz (mm), (SD)	3.90 (3.29)	2.70 (1.26)	3.00 (3.82)	4.50 (3.52)	4.20 (2.47)	2.70 (3.25)	
**Thyroid**							
DSC, (SD)	0.73 (0.11)	0.77 (0.06)	0.73 (0.07)	0.81 (0.05)	0.82 (0.06)	0.82 (0.05)	^
Sensitivity, (SD)	0.79 (0.15)	0.81 (0.10)	0.79 (0.08)	0.86 (0.08)	0.84 (0.05)	0.84 (0.04)	
IncI, (SD)	0.69 (0.11)	0.75 (0.10)	0.70 (0.11)	0.78 (0.10)	0.81 (0.10)	0.80 (0.07)	°
ΔV (%), (SD)	14.86 (23.01)	10.34 (28.54)	15.70 (26.54)	14.00 (25.25)	5.72 (17.65)	5.98 (9.27)	°
Δx (mm), (SD)	0.98 (0.82)	1.26 (1.51)	1.48 (1.37)	0.50 (0.00)	0.70 (0.98)	1.56 (1.93)	
Δy (mm), (SD)	1.18 (1.06)	0.98 (0.90)	1.68 (2.12)	0.70 (0.45)	0.40 (0.42)	0.60 (0.55)	
Δz (mm), (SD)	3.00 (2.37)	2.10 (2.28)	1.50 (1.50)	2.70 (2.47)	1.20 (1.96)	2.10 (1.34)	
**Masticator space right**							
DSC, (SD)	0.80 (0.04)	0.82 (0.03)	0.80 (0.03)	0.85 (0.02)	0.84 (0.04)	0.84 (0.02)	°
Sensitivity, (SD)	0.79 (0.07)	0.84 (0.03)	0.80 (0.06)	0.83 (0.07)	0.91 (0.02)	0.86 (0.03)	° + =
IncI, (SD)	0.84 (0.10)	0.80 (0.03)	0.80 (0.02)	0.86 (0.05)	0.79 (0.07)	0.82 (0.02)	+ ^
ΔV (%), (SD)	−3.52 (21.72)	4.74 (3.43)	−0.62 (6.99)	−3.16 (12.28)	16.28 (12.24)	5.14 (2.48)	° =
Δx (mm), (SD)	0.82 (0.58)	1.00 (0.5)	0.82 (0.58)	0.50 (0.71)	0.90 (0.82)	0.82 (0.30)	
Δy (mm), (SD)	1.12 (0.69)	1.20 (0.99)	1.20 (1.29)	1.02 (0.36)	1.78 (1.85)	1.30 (1.19)	
Δz (mm), (SD)	3.30 (1.64)	4.80 (5.13)	5.40 (4.70)	2.70 (0.67)	3.00 (3.52)	2.70 (1.64)	
**Masticator Space left**							
DSC, (SD)	0.79 (0.05)	0.81 (0.05)	0.77 (0.05)	0.86 (0.02)	0.86 (0.03)	0.85 (0.02)	* # °
Sensitivity, (SD)	0.72 (0.08)	0.79 (0.08)	0.73 (0.09)	0.82 (0.05)	0.89 (0.03)	0.86 (0.04)	* ° + ^
IncI, (SD)	0.89 (0.05)	0.85 (0.01)	0.82 (0.01)	0.90 (0.02)	0.83 (0.07)	0.84 (0.03)	° + ^
ΔV (%), (SD)	−18.64 (12.32)	−7.02 (10.16)	−10.58 (12.18)	−8.30 (5.83)	8.26 (10.51)	3.00 (5.82)	° + ^
Δx (mm), (SD)	0.84 (1.34)	1.02 (0.65)	1.02 (0.82)	1.040 (0.96)	0.92 (0.58)	0.62 (0.69)	
Δy (mm), (SD)	2.26 (1.85)	1.76 (1.27)	2.12 (0.80)	1.820 (1.08)	2.52 (2.76)	1.94 (1.37)	
Δz (mm), (SD)	3.00 (2.60)	6.00 (5.09)	6.00 (5.91)	2.40 (0.82)	3.00 (4.37)	2.40 (1.34)	
**Mandible**							
DSC, (SD)	0.89 (0.02)	0.86 (0.02)	0.84 (0.02)	0.90 (0.02)	0.89 (0.02)	0.89 (0.02)	*
Sensitivity, (SD)	0.87 (0.05)	0.86 (0.04)	0.82 (0.04)	0.88 (0.04)	0.92 (0.03)	0.92 (0.03)	+ ^
IncI, (SD)	0.92 (0.02)	0.86 (0.05)	0.86 (0.06)	0.92 (0.02)	0.86 (0.05)	0.87 (0.05)	* # + ^
ΔV (%), (SD)	−4.76 (7.12)	−0.48 (9.51)	−3.60 (12.00)	−3.88 (6.02)	7.06 (9.05)	7.02 (8.78)	
Δx (mm), (SD)	0.30 (0.27)	0.60 (0.42)	0.50 (0.35)	0.30 (0.27)	0.70 (0.57)	0.30 (0.27)	
Δy (mm), (SD)	0.30 (0.27)	0.40 (0.22)	0.50 (0.35)	0.30 (0.27)	0.30 (0.27)	0.20 (0.27)	
Δz (mm), (SD)	0.60 (0.82)	0.30 (0.67)	1.20 (1.26)	0.90 (0.82)	0.60 (0.82)	0.60 (0.82)	
**Spinal cord**							
DSC, (SD)	0.70 (0.10)	0.81 (0.05)	0.78 (0.06)	0.84 (0.02)	0.87 (0.01)	0.86 (0.01)	*
Sensitivity, (SD)	0.60 (0.14)	0.78 (0.10)	0.75 (0.08)	0.78 (0.05)	0.87 (0.03)	0.88 (0.04)	* #
IncI, (SD)	0.89 (0.07)	0.86 (0.07)	0.83 (0.08)	0.91 (0.05)	0.87 (0.02)	0.84 (0.03)	
ΔV (%), (SD)	−32.46 (19.30)	−9.00 (16.93)	−8.40 (14.92)	−13.18 (9.26)	0.66 (4.55)	5.52 (8.34)	* # + ^
Δx (mm), (SD)	0.80 (0.45)	0.60 (0.22)	0.92 (0.66)	1.00 (0.80)	0.90 (0.42)	0.30 (0.27)	
Δy (mm), (SD)	3.62 (6.98)	4.88 (7.20)	2.78 (3.96)	0.92 (0.43)	0.32 (0.72)	0.62 (0.59)	
Δz (mm), (SD)	7.80 (11.74)	7.20 (12.79)	6.00 (10.92)	0.60 (0.82)	0.30 (0.67)	0.00 (0.00)	
**Parotid gland right**							
DSC, (SD)	0.78 (0.08)	0.79 (0.07)	0.73 (0.08)	0.80 (0.06)	0.82 (0.05)	0.80 (0.05)	# ° =
Sensitivity, (SD)	0.79 (0.13)	0.82 (0.12)	0.79 (0.16)	0.82 (0.12)	0.86 (0.10)	0.84 (0.10)	+
IncI, (SD)	0.80 (0.11)	0.77 (0.09)	0.71 (0.08)	0.80 (0.09)	0.79 (0.07)	0.79 (0.10)	#
ΔV (%), (SD)	−1.06 (21.92)	8.96 (21.67)	13.02 (29.54)	4.40 (22.94)	10.40 (18.23)	9.14 (23.61)	*
Δx (mm), (SD)	1.50 (1.00)	1.62 (1.24)	2.02 (1.06)	1.96 (1.79)	1.62 (1.24)	1.94 (1.19)	
Δy (mm), (SD)	6.46 (7.06)	5.64 (5.66)	6.58 (6.25)	6.4 (7.11)	4.32 (6.34)	5.76 (7.04)	
Δz (mm), (SD)	2.40 (0.82)	2.40 (0.82)	3.60 (2.51)	1.80 (1.26)	1.50 (1.06)	1.20 (1.64)	
**Parotid gland left**							
DSC, (SD)	0.79 (0.07)	0.78 (0.08)	0.73 (0.04)	0.80 (0.07)	0.81 (0.06)	0.81 (0.05)	°
Sensitivity, (SD)	0.77 (0.11)	0.79 (0.11)	0.77 (0.05)	0.79 (0.11)	0.83 (0.09)	0.85 (0.07)	^
IncI, (SD)	0.82 (0.07)	0.77 (0.11)	0.69 (0.04)	0.82 (0.09)	0.80 (0.11)	0.78 (0.08)	#
ΔV (%), (SD)	−5.84 (17.41)	4.44 (20.36)	12.96 (4.75)	−1.88 (21.29)	6.54 (21.64)	9.78 (14.61)	*
Δx (mm), (SD)	2.00 (1.46)	2.46 (1.23)	2.12 (1.43)	2.12 (1.62)	2.08 (1.46)	3.08 (1.75)	^
Δy (mm), (SD)	1.68 (2.21)	2.60 (1.37)	2.12 (1.76)	1.38 (1.60)	0.78 (1.21)	1.38 (2.00)	
Δz (mm), (SD)	3.60 (2.73)	3.30 (2.68)	4.50 (1.50)	2.70 (1.96)	3.60 (2.73)	2.70 (2.23)	
**Cochlea right**							
DSC, (SD)	0.63 (0.13)	0.63 (0.17)	0.46 (0.16)	0.80 (0.03)	0.77 (0.10)	0.69 (0.07)	# ° ^
Sensitivity, (SD)	0.59 (0.17)	0.68 (0.24)	0.42 (0.13)	0.77 (0.09)	0.84 (0.11)	0.80 (0.10)	# °
IncI, (SD)	0.71 (0.17)	0.64 (0.23)	0.55 (0.25)	0.88 (0.09)	0.72 (0.07)	0.64 (0.14)	# + ^
ΔV (%), (SD)	−5.28 (6.84)	16.94 (54.41)	−15.74 (23.04)	−7.36 (17.40)	17.9 (21.82)	33.98 (49.56)	+
Δx (mm), (SD)	0.90 (0.42)	0.72 (0.47)	1.28 (1.11)	0.42 (0.24)	0.62 (0.69)	0.72 (0.88)	
Δy (mm), (SD)	1.38 (1.77)	1.38 (1.20)	1.60 (1.90)	0.40 (0.42)	0.30 (0.27)	0.72 (0.30)	
Δz (mm), (SD)	0.50 (0.71)	0.90 (0.82)	2.40 (2.01)	0.40 (0.65)	0.30 (0.67)	0.90 (0.82)	
**Cochlea left**							
DSC, (SD)	0.52 (0.16)	0.69 (0.10)	0.59 (0.07)	0.78 (0.07)	0.78 (0.04)	0.72 (0.07)	°
Sensitivity, (SD)	0.45 (0.22)	0.69 (0.13)	0.58 (0.07)	0.69 (0.09)	0.81 (0.05)	0.75 (0.08)	* +
IncI, (SD)	0.87 (0.10)	0.70 (0.15)	0.63 (0.13)	0.92 (0.05)	0.76 (0.09)	0.72 (0.16)	* # + ^
ΔV (%), (SD)	−51.04 (29.55)	6.06 (30.04)	−5.40 (13.70)	−23.32 (10.44)	10.24 (12.62)	11.50 (34.88)	* + ^
Δx (mm), (SD)	1.00 (0.50)	0.60 (0.42)	1.00 (0.71)	0.30 (0.27)	0.40 (0.22)	0.50 (0.50)	
Δy (mm), (SD)	0.40 (0.42)	0.40 (0.42)	0.90 (0.55)	0.60 (0.55)	0.20 (0.45)	1.00 (0.50)	=
Δz (mm), (SD)	0.90 (0.82)	1.00 (1.28)	1.00 (1.28)	0.30 (0.67)	1.20 (1.26)	0.40 (0.65)	
**Brain stem**							
DSC, (SD)	0.80 (0.07)	0.81 (0.11)	0.77 (0.15)	0.88 (0.02)	0.88 (0.03)	0.89 (0.03)	
Sensitivity, (SD)	0.74 (0.09)	0.76 (0.17)	0.73 (0.22)	0.86 (0.04)	0.86 (0.08)	0.89 (0.03)	
IncI, (SD)	0.89 (0.06)	0.89 (0.03)	0.85 (0.03)	0.90 (0.02)	0.90 (0.03)	0.88 (0.04)	
ΔV (%), (SD)	−17.50 (8.23)	−14.36 (19.96)	−13.68 (26.28)	−4.54 (6.22)	−4.00 (10.88)	1.44 (1.75)	^
Δx (mm), (SD)	0.70 (0.57)	1.32 (0.78)	1.12 (0.69)	0.60 (0.22)	0.92 (0.86)	1.04 (1.02)	
Δy (mm), (SD)	1.40 (0.82)	1.76 (0.80)	1.88 (0.74)	0.68 (0.82)	0.90 (1.34)	1.10 (0.67)	
Δz (mm), (SD)	2.40 (3.29)	3.00 (3.82)	4.20 (4.67)	0.90 (0.82)	0.90 (1.34)	0.30 ()	
**Esophagus**							
DSC, (SD)	0.63 (0.10)	0.65 (0.15)	0.64 (0.13)	0.83 (0.05)	0.84 (0.06)	0.83 (0.06)	
Sensitivity, (SD)	0.57 (0.15)	0.57 (0.19)	0.63 (0.17)	0.85 (0.06)	0.87 (0.04)	0.88 (0.02)	
IncI, (SD)	0.76 (0.12)	0.79 (0.09)	0.67 (0.11)	0.82 (0.10)	0.82 (0.11)	0.79 (0.10)	°
ΔV (%), (SD)	−21.68 (29.94)	−27.04 (26.22)	−5.92 (23.75)	5.62 (18.34)	8.26 (18.26)	12.04 (12.37)	
Δx (mm), (SD)	1.12 (0.42)	1.38 (1.05)	1.54 (0.86)	0.60 (0.65)	0.80 (0.57)	1.00 (0.79)	
Δy (mm), (SD)	3.84 (5.83)	4.54 (6.48)	3.66 (3.77)	0.82 (0.84)	1.12 (0.76)	0.72 (0.79)	
Δz (mm), (SD)	7.80 (11.64)	7.50 (12.68)	6.60 (10.64)	1.50 (1.06)	1.20 (1.26)	1.20 (1.26)	

For automatic segmentation: *AA vs. MA: p<0.043, #AA vs. VA: p<0.043, °MA vs. VA: p<0.043.

For automatic segmentation+ manual correction: +AM vs. MM: p<0.043, ^AM vs. VM: p<0.043, =MM vs. VM: p<0.043.

*Abbreviations: CTV* = Clinical target volume; *SD* = Standard deviation, *DSC* = DICE similarity coefficient, *IncI* = inclusiveness index, *AA* = Abas Automatic, *MA* = MiM Automatic, *VA* = Velocity Automatic, *AM* = Abas Manual Correction, *MM* = MiM Manual Correction, *VM* = Velocity Manual Correction.

Finally, in the mesothelioma cases (Table 4), the sensitivity index was usually higher for ABAS, before manual correction, and for VelocityAI after manual correction. Regarding the IncI index statistically significant differences are present in both automatic and manually corrected contours: MIM usually resulted to be the best software in both cases. For the automatic contours, the ΔV ranged from about +30% for the esophagus, to −10% for the intestine; these differences usually decreased after ACMC, but sometimes remained high (i.e. CTV, esophagus, and spinal cord).

**Table 4:** Mean values and standard deviations of parameters that evaluate the contours generated by the three software, before and after the manual correction, for each organ of the mesothelioma patients

	**AA**	**MA**	**VA**	**AM**	**MM**	**VM**	**p value**
**CTV**							
DSC, (SD)	0.85 (0.02)	0.86 (0.02)	0.85 (0.02)	0.85 (0.02)	0.94 (0.04)	0.91 (0.02)	+ ^
Sensitivity. (SD)	0.92 (0.03)	0.90 (0.02)	0.91 (0.02)	0.92 (0.03)	0.93 (0.03)	0.94 (0.03)	* ° ^
IncI. (SD)	0.79 (0.02)	0.82 (0.02)	0.80 (0.02)	0.80 (0.02)	0.95 (0.06)	0.87 (0.02)	* ° + ^ =
ΔV (%). (SD)	15.54 (1.53)	9.26 (2.06)	13.48 (2.45)	14.90 (1.45)	−1.78 (4.95)	8.10 (3.04)	* ° + ^ =
Δx (mm). (SD)	3.42 (4.20)	3.42 (2.79)	4.10 (2.24)	4.20 (4.24)	0.98 (1.24)	3.40 (2.51)	=
Δy (mm). (SD)	3.82 (3.60)	3.92 (3.61)	3.52 (3.90)	3.54 (3.69)	1.78 (1.56)	2.36 (1.51)	
Δz (mm). (SD)	4.00 (4.87)	4.00 (4.87)	4.50 (4.80)	4.00 (3.35)	0.50 (1.12)	0.00 (0.00)	^
**Contra lateral lung**							
DSC. (SD)	0.95 (0.03)	0.94 (0.03)	0.94 (0.02)	0.97 (0.01)	0.98 (0.01)	0.99 (0.01)	^ =
Sensitivity. (SD)	0.93 (0.05)	0.90 (0.06)	0.92 (0.04)	0.97 (0.01)	0.97 (0.01)	0.99 (0.01)	° ^ =
IncI. (SD)	0.97 (0.02)	0.99 (0.01)	0.96 (0.020)	0.97 (0.02)	0.98 (0.03)	0.99 (0.01)	* °
ΔV (%). (SD)	−4.66 (5.16)	−8.68 (6.59)	−4.16 (5.43)	0.14 (3.36)	−1.24 (3.51)	−0.52 (0.98)	* °
Δx (mm). (SD)	0.70 (0.67)	0.40 (0.55)	0.88 (1.43)	1.48 (2.22)	0.60 (0.42)	0.00 (0.00)	
Δy (mm). (SD)	2.46 (2.59)	2.46 (2.59)	2.24 (2.50)	1.30 (0.67)	0.90 (0.96)	0.00 (0.00)	^
Δz (mm). (SD)	7.50 (7.07)	7.50 (7.07)	10.00 (7.91)	1.88 (3.75)	1.88 (3.75)	0.00 (0.00)	
**Esophagus**							
DSC. (SD)	0.68 (0.08)	0.67 (0.08)	0.62 (0.03)	0.69 (0.08)	0.83 (0.12)	0.76 (0.02)	# °
Sensitivity. (SD)	0.78 (0.08)	0.69 (0.10)	0.73 (0.09)	0.79 (0.08)	0.80 (0.13)	0.84 (0.07)	* #
IncI. (SD)	0.61 (0.13)	0.67 (0.13)	0.54 (0.07)	0.62 (0.13)	0.86 (0.12)	0.70 (0.06)	* ° + =
ΔV (%). (SD)	32.92 (31.51)	6.80 (25.74)	37.98 (28.67)	33.46 (32.52)	−5.98 (11.80)	21.22 (17.13)	* ° + =
Δx (mm). (SD)	3.82 (2.46)	2.36 (1.84)	2.92 (2.49)	3.34 (1.87)	1.30 (0.76)	1.40 (0.55)	^
Δy (mm). (SD)	2.74 (2.78)	2.54 (2.84)	2.26 (2.33)	2.74 (2.77)	1.18 (1.81)	2.16 (2.92)	
Δz (mm). (SD)	7.50 (7.29)	8.00 (6.94)	7.00 (6.47)	7.50 (7.29)	5.00 (7.32)	5.50 (7.37)	+
**Heart**							
DSC. (SD)	0.88 (0.03)	0.87 (0.05)	0.88 (0.05)	0.90 (0.01)	0.94 (0.01)	0.95 (0.01)	+ ^
Sensitivity. (SD)	0.86 (0.09)	0.82 (0.10)	0.86 (0.10)	0.89 (0.06)	0.93 (0.03)	0.94 (0.02)	* ° ^
IncI. (SD)	0.91 (0.04)	0.93 (0.04)	0.90 (0.03)	0.91 (0.05)	0.96 (0.04)	0.96 (0.02)	* ^
ΔV (%). (SD)	−4.48 (14.19)	−11.40 (13.81)	−4.58 (14.07)	−1.50 (11.80)	−2.30 (6.80)	−2.02 (4.08)	* °
Δx (mm). (SD)	1.26 (1.04)	1.18 (0.80)	1.20 (0.45)	1.38 (0.99)	0.80 (0.45)	1.58 (0.93)	
Δy (mm). (SD)	1.78 (1.22)	1.58 (1.26)	1.76 (1.48)	1.86 (1.25)	1.08 (1.10)	1.28 (0.72)	
Δz (mm). (SD)	2.50 (1.77)	3.50 (2.85)	3.50 (2.24)	3.00 (2.09)	1.50 (1.37)	2.00 (2.09)	
**Intestine**							
DSC. (SD)	0.77 (0.13)	0.74 (0.15)	0.77 (0.13)	0.87 (0.03)	0.93 (0.04)	0.93 (0.02)	+ ^
Sensitivity. (SD)	0.75 (0.21)	0.68 (0.21)	0.74 (0.21)	0.90 (0.05)	0.90 (0.06)	0.95 (0.01)	* ° ^
IncI. (SD)	0.85 (0.09)	0.86 (0.08)	0.84 (0.10)	0.86 (0.07)	0.97 (0.03)	0.92 (0.04)	* ° + ^ =
ΔV (%). (SD)	−9.54 (30.30)	−19.36 (29.33)	−9.7 (31.47)	5.46 (14.22)	−7.48 (5.73)	2.98 (5.13)	* ° + =
Δx (mm). (SD)	2.94 (1.36)	4.90 (4.77)	4.52 (5.51)	2.08 (1.21)	1.58 (1.05)	1.08 (1.10)	^
Δy (mm). (SD)	4.12 (4.23)	4.20 (4.05)	4.70 (3.83)	1.86 (1.55)	1.48 (0.67)	1.86 (2.54)	
Δz (mm). (SD)	11.50 (8.40)	12.00 (7.37)	12.00 (7.16)	3.00 (4.11)	0.00 (0.00)	1.00 (1.37)	
**Liver**							
DSC. (SD)	0.93 (0.02)	0.93 (0.03)	0.90 (0.01)	0.93 (0.03)	0.97 (0.02)	0.95 (0.02)	# ° +
Sensitivity. (SD)	0.92 (0.02)	0.90 (0.03)	0.92 (0.03)	0.93 (0.03)	0.95 (0.03)	0.96 (0.02)	^
IncI. (SD)	0.93 (0.03)	0.95 (0.04)	0.88 (0.02)	0.93 (0.03)	0.98 (0.02)	0.94 (0.03)	* # ° + =
ΔV (%). (SD)	−0.80 (2.10)	−5.08 (2.16)	5.44 (4.93)	0.00 (2.29)	−2.96 (2.41)	2.26 (4.28)	* ° + =
Δx (mm). (SD)	3.24 (2.53)	2.64 (1.81)	3.80 (2.26)	2.16 (1.90)	0.60 (0.65)	0.70 (0.45)	
Δy (mm). (SD)	1.28 (1.11)	1.56 (1.09)	1.38 (0.70)	1.18 (1.27)	0.60 (0.55)	1.00 (0.70)	
Δz (mm). (SD)	1.50 (2.24)	3.50 (2.85)	4.50 (3.26)	2.50 (1.77)	1.00 (1.37)	1.00 (1.37)	
**Kidney left**							
DSC. (SD)	0.89 (0.02)	0.87 (0.03)	0.81 (0.06)	0.91 (0.01)	0.95 (0.02)	0.92 (0.02)	* # ° =
Sensitivity. (SD)	0.88 (0.05)	0.84 (0.06)	0.81 (0.12)	0.93 (0.02)	0.92 (0.03)	0.91 (0.02)	* #
IncI. (SD)	0.89 (0.02)	0.90 (0.03)	0.82 (0.04)	0.88 (0.02)	0.98 (0.03)	0.93 (0.04)	* # ° + =
ΔV (%). (SD)	−0.98 (7.21)	−7.40 (8.71)	−1.40 (15.64)	4.90 (4.36)	−6.08 (3.41)	−1.10 (5.61)	* +
Δx (mm). (SD)	0.90 (0.74)	1.10 (0.65)	1.30 (0.57)	0.80 (0.57)	0.50 (0.50)	1.00 (0.79)	
Δy (mm). (SD)	0.60 (0.42)	0.70 (0.84)	1.18 (1.11)	0.40 (0.22)	0.20 (0.27)	0.50 (0.36)	
Δz (mm). (SD)	2.00 (1.12)	4.00 (2.85)	6.50 (5.18)	1.50 (1.37)	0.00 (0.00)	0.00 (0.00)	
**Kidney right**							
DSC. (SD)	0.89 (0.02)	0.86 (0.04)	0.83 (0.05)	0.91 (0.02)	0.95 (0.02)	0.93 (0.01)	#
Sensitivity. (SD)	0.88 (0.04)	0.83 (0.06)	0.81 (0.10)	0.93 (0.03)	0.91 (0.03)	0.92 (0.02)	#
IncI. (SD)	0.90 (0.05)	0.91 (0.05)	0.86 (0.09)	0.89 (0.05)	0.99 (0.02)	0.94 (0.02)	# ° + ^ =
ΔV (%). (SD)	−1.92 (9.47)	−8.68 (8.81)	−4.30 (17.24)	4.28 (7.74)	−7.72 (1.43)	−2.46 (3.08)	+ ^ =
Δx (mm). (SD)	1.46 (1.13)	1.06 (1.24)	1.10 (0.74)	0.80 (0.76)	0.60 (0.42)	0.50 (0.71)	
Δy (mm). (SD)	0.60 (0.55)	0.70 (0.45)	1.08 (1.70)	0.60 (0.55)	0.50 (0.35)	0.80 (0.57)	
Δz (mm). (SD)	3.50 (2.85)	3.00 (4.11)	5.00 (6.37)	1.00 (1.37)	0.50 (1.12)	0.50 (1.12)	
**Spinal cord**							
DSC. (SD)	0.71 (0.07)	0.69 (0.08)	0.74 (0.06)	0.73 (0.04)	0.83 (0.02)	0.86 (0.05)	# ° ^
Sensitivity. (SD)	0.65 (0.15)	0.61 (0.16)	0.74 (0.16)	0.69 (0.12)	0.72 (0.18)	0.85 (0.11)	* # ° ^
IncI. (SD)	0.82 (0.15)	0.85 (0.13)	0.78 (0.12)	0.83 (0.14)	1.00 (0.00)	0.88 (0.06)	* # ° + =
ΔV (%). (SD)	−15.72 (32.61)	−24.9 (31.50)	−0.72 (34.59)	−12.86 (31.11)	−27.76 (18.01)	−3.02 (16.70)	* # ° =
Δx (mm). (SD)	1.00 (0.61)	0.80 (0.27)	0.40 (0.42)	1.00 (0.61)	0.30 (0.45)	0.40 (0.65)	
Δy (mm). (SD)	4.70 (5.93)	4.40 (4.90)	4.02 (4.06)	4.70 (5.93)	0.80 (0.67)	0.2 (0.27)	
Δz (mm). (SD)	22.00 (23.21)	22.00 (22.87)	21.50 (21.84)	9.00 (8.94)	3.00 (4.11)	1.00 (2.24)	
**Spleen**							
DSC. (SD)	0.92 (0.01)	0.90 (0.03)	0.84 (0.04)	0.93 (0.01)	0.95 (0.02)	0.94 (0.01)	# ° =
Sensitivity. (SD)	0.90 (0.00)	0.86 (0.03)	0.88 (0.02)	0.92 (0.01)	0.92 (0.02)	0.94 (0.01)	^
IncI. (SD)	0.93 (0.02)	0.96 (0.03)	0.80 (0.06)	0.94 (0.01)	0.99 (0.02)	0.93(0.03)	* # ° +
ΔV (%). (SD)	−3.85 (2.04)	−10.38 (2.89)	9.90 (10.45)	−3.00 (1.12)	−6.73 (1.16)	1.18 (4.04)	* +
Δx (mm). (SD)	0.75 (0.65)	0.88 (0.48)	1.00 (0.41)	1.48 (0.78)	0.50 (0.00)	0.75 (0.64)	
Δy (mm). (SD)	2.58 (2.89)	2.95 (2.61)	3.58 (3.49)	1.73 (1.51)	1.00 (0.58)	1.75 (0.29)	
Δz (mm). (SD)	2.50 (2.04)	1.88 (2.93)	3.75 (2.23)	1.25 (1.44)	0.00 (0.00)	1.88 (2.39)	

For automatic segmentation: *AA vs. MA: p<0.043, #AA vs. VA: p<0.043, °MA vs. VA: p<0.043.

For automatic segmentation+ manual correction: +AM vs. MM: p<0.043, ^AM vs. VM: p<0.043, =MM vs. VM: p<0.043.

*Abbreviations: CTV* = Clinical target volume; *SD* = Standard deviation, *DSC* = DICE similarity coefficient, *IncI* = inclusiveness index, *AA* = Abas Automatic, *MA* = MiM Automatic, *VA* = Velocity Automatic, *AM* = Abas Manual Correction, *MM* = MiM Manual Correction, *VM* = Velocity Manual Correction.

**Figure 2:**
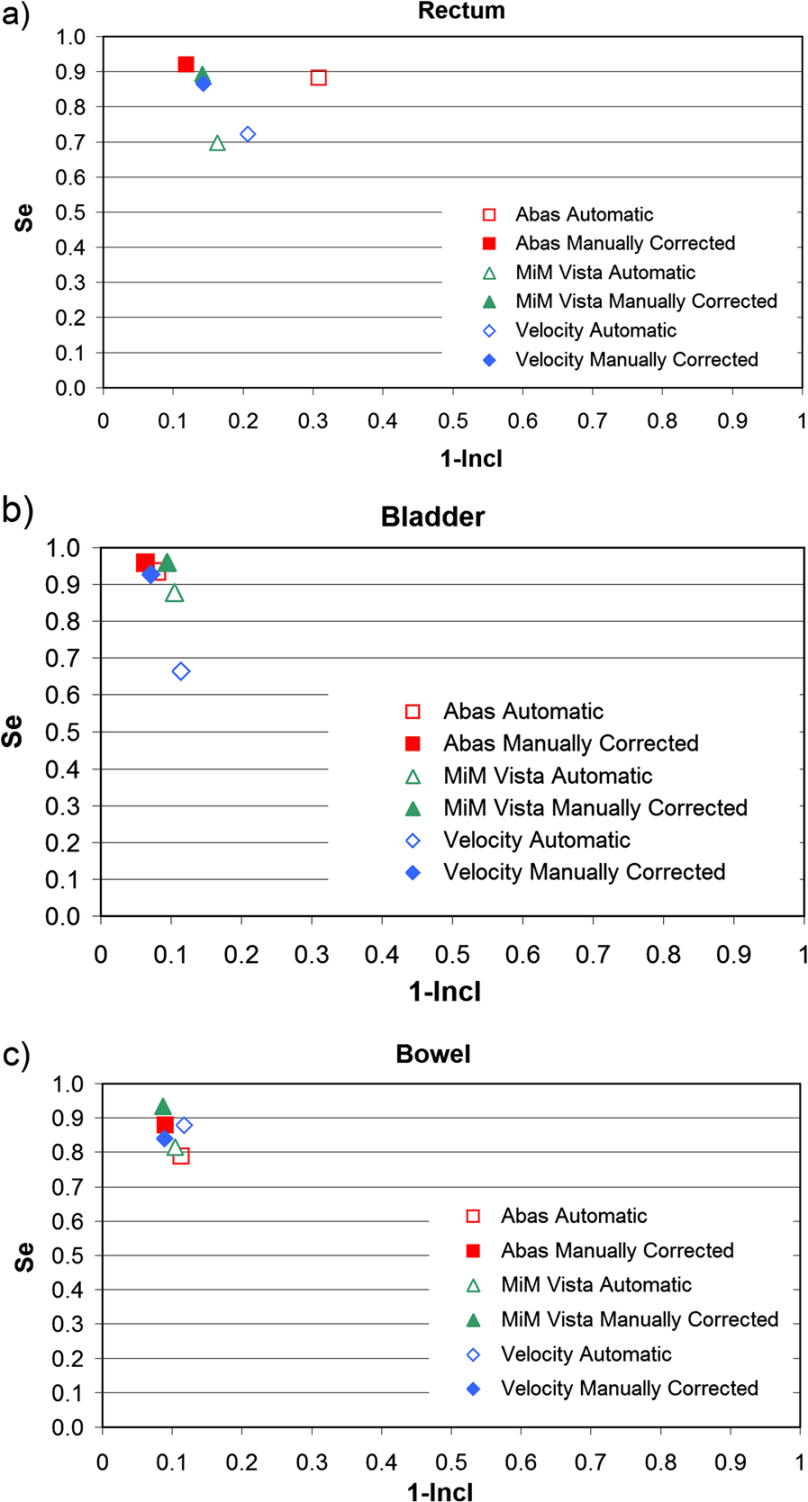
**mROC analysis of the performances of three software solutions evaluated for some OARs of prostate patients: a) rectum, b) bladder, and c) bowel.** Automatic segmentation and automatic segmentation + correction were compared to the manual contours from scratch.

**Figure 3:**
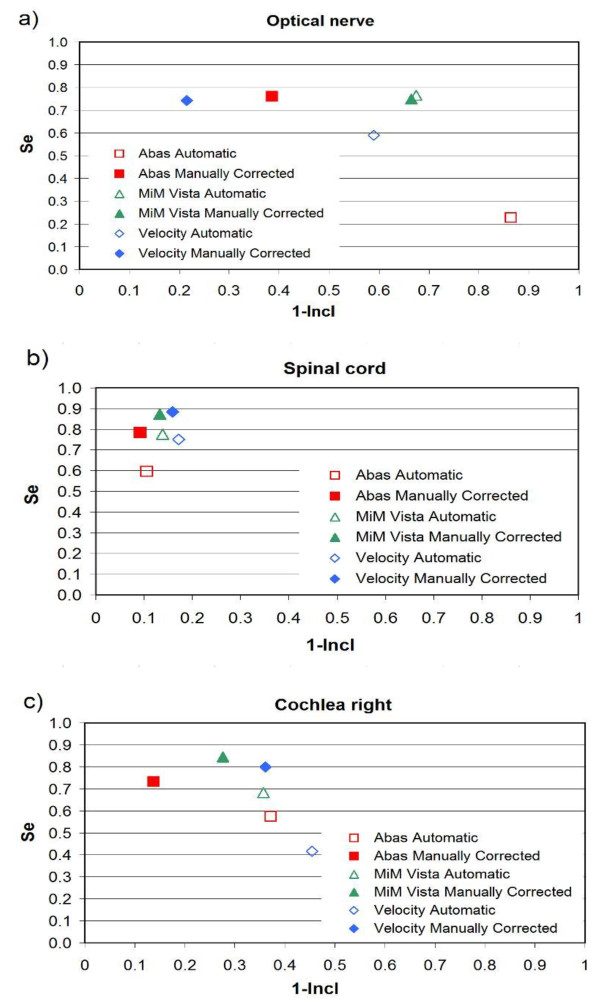
**mROC analysis of the performances of three software solutions evaluated in H&N patients: a) optical nerve, b) spinal cord, and c) cochlea right.** Automatic segmentation and automatic segmentation + correction were compared to the manual contours from scratch.

**Figure 4:**
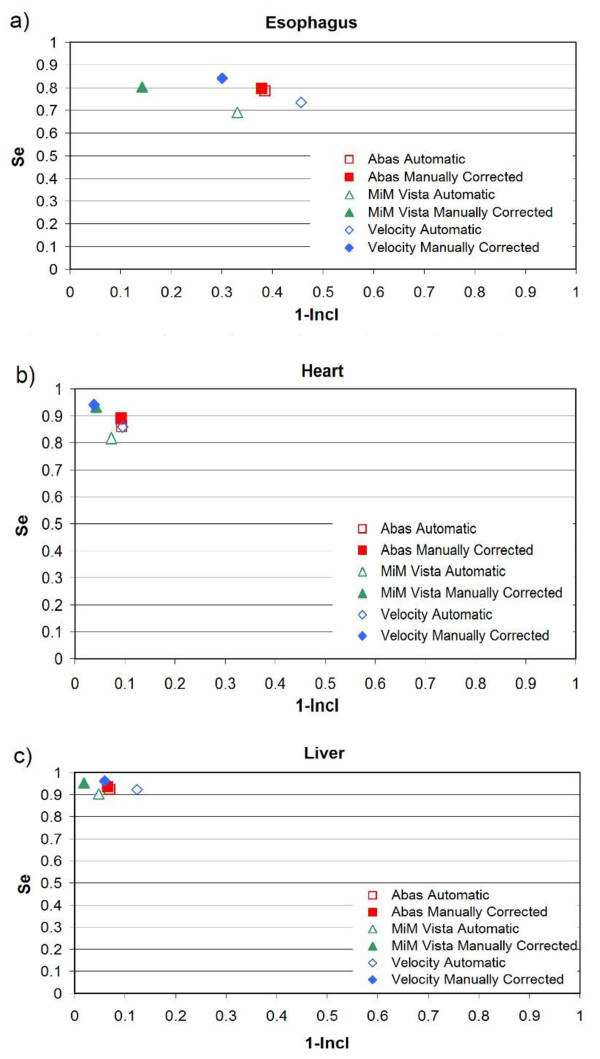
**mROC analysis of the performances of three software solutions evaluated in mesothelioma patients: a) esophagus, b) heart, and c) liver.** Automatic segmentation and automatic segmentation + correction were compared to the manual contours from scratch.

Figures 2, 3 and 4 show the mROC analysis of the performance of automatic segmentation compared to manual correction after automatic segmentation. The mROC curves for the selected OARs exhibited the same behaviour: all the points improved with manual correction (i.e. they moved to the upper left corner of the mROC space), but under our clinical conditions some discrepancies still remain compared to the reference structure.

Regarding the automatic re contouring of the tumor, we can summarize that for prostate patients the DSC index improved after manual correction, but it still remained below 0.9; in H&N cases, the DSC index improved after manual correction for CTV1 (but still remained below 0.8) and almost didn’t vary for CTV2. In the mesothelioma patients, the DSC index improved, for the three software solutions, from an average value of 0.85 before manual correction to 0.9, with MIM performing slightly better than the other two software solutions.

Furthermore due to the low number of patients examined, applying the Holm-Bonferroni method, none of the difference between the software solutions would have been statistically significant. That is why we reported in our Tables the *p* values obtained with the Wilcoxon test.

## Discussion

The need to replan and adapt treatment for internal anatomy variations due to tumor shrinkage and shape deformation [1,3] has increased over the years in order to make better use of highly conformal treatment techniques. However, this modality is very time-consuming. In order to reduce the commitment of medical staff in targets and ROIs delineation and modification, systems for the AC have been increasingly developed. The use of atlas-based tools to delineate OARs for cancer sites including H&N [11,14], breast [15], endometrium [16] and prostate [17] have shown to reduce volume delineation variability and the total time required to contour. In this study, we compared three different commercial software solutions for atlas-based autocontouring through a comparison with manual delineation of target and OAR in three tumor sites. For the purpose of this study, contours of manually-generated VOIs on pCT were taken as a reference atlas. These were then compared to the VOIs (contours) automatically generated by A, M and V, and successively corrected manually. This procedure has proved to be time saving although the AC must be re-checked and corrected manually by physicians: on average, about 40 minutes were saved for the HRPCa, one hour for the H&N patients, and 20 minutes for the MPM.

**Figure 5:**
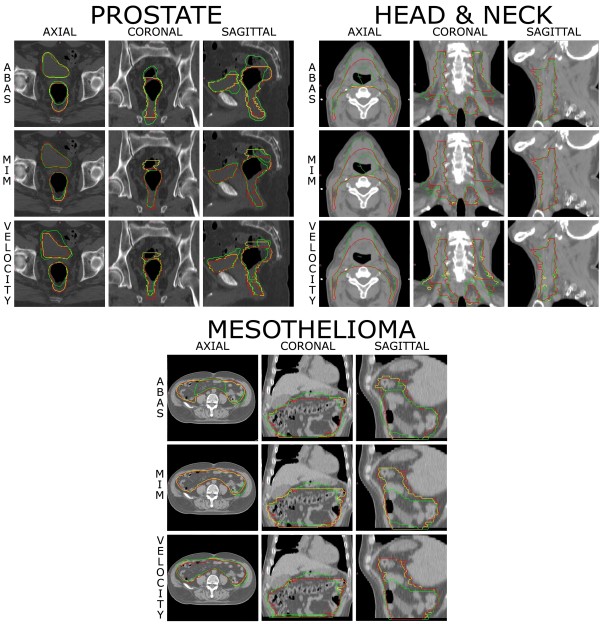
Patient manually contoured from scratch (red), automatically segmented (green) by the three software solutions and corrected after automatic segmentation (yellow) for a HRPCa, a H&N case, and a MPM.

Regarding prostate cases, the auto-segmentation module faces the same problems as the clinicians when drawing the prostate: a) in the cranial direction there is poor or no contrast on the CT between the base of the prostate and the bladder, b) in the caudal direction, there is poor or no contrast between the apex of the prostate and the rectum. As in the correction/replanning of the prostate plans, the volumes that needed the most corrections were the rectum, the CTV and the bowel. The volumes closer to the reference volumes were the femoral heads and the bladder. It was noted that the most cranial and caudal slices of all volumes underwent more changes leading to greater intraobserver variability. This was especially true for certain organs, such as the rectum (Figure 5) (i.e. we also found that after manual correction, the ΔV and Δz parameter can remain significant for some organs). For the Vref, the rectum was contoured according to the guidelines [9] and for the AC, these anatomical limits were not always respected. This may lead to the intraobserver variability: the AC could bring us to correct a contour that is misleading from the start for the physician. The placement of a rectal balloon and a strict protocol for bladder filling could help the automatic recontouring process for the prostate patient.

With regards to the H&N cases, the first consideration is that the re-planning CT was performed without contrast media; surely having contrast media would have been helpful for the physician for target delineation and probably for the three software packages too. Nonetheless results, both in terms of time and contouring accuracy are good and promising. All three software solutions significantly reduce the time needed to replan the VOIs in comparison to the time needed to replan the same VOIs from scratch (Vref). Indeed, both the automatic contouring time, the manual correction time and their sum are statistically shorter than the Vref contouring time. Each software allows 1 hour to be saved, which is undoubtedly relevant in daily clinical activity. The significant differences found amongst the times provided by the three software packages can be explained in part with the fact that the referring contouring physician for the H&N area had been using one of them in his clinical practice during the months preceding the analysis. When evaluating quality according to the established parameters, ΔV, Δx,y,z, DSC, sensitivity and inclusiveness indexes, VOIs generated with the AC and VOIs manually corrected from AC compares favourably with their corresponding Vref VOIs. Indeed even lower scores of the quality indexes are in an acceptable range. As for the prostate cases, we found a volume variation between the Vref VOIs and the automatic generated manually corrected VOIs in particular for CTVs, for organs with not-precisely-defined boundaries such as the superior pharynx and for organs of small volume such as the cochlea. This intra-observer variability is a well known phenomenon of the radiotherapy planning more evident when there are no anatomical points of reference. As for rectum, another explanation could be that automatic contour propagation produce contours which somehow "suggest" to the human observer incorrect contour shape. Variation in the position of center of mass is particularly evident for the z axis both for automatically generated and manually corrected but it is limited to the length of a couple of slices. As expected, scores of DSC, sensitivity and inclusiveness indexes for ACMC contours are better than the automatically generated corresponding ones, pointing out the necessity of correction from a physician of the automatically generated VOIs.

In the mesothelioma cases the bowel required some work to be re-contoured manually particularly in the most cranial and caudal slices. Moreover, thoracic cavity showed some differences probably for a different content of air cavities, requesting some more manual interventions.

The accuracy evaluated with sensitivity, inclusiveness and DSC indexes, and the other volumetric parameters, show that none of the three software solutions always perform better: depending on the VOI, parameter and type of cancer considered, from time to time one software can be better than another. More importantly, a statistically significant difference between the software solutions does not lead to a clinically relevant difference. Looking at the data reported in our tables (Tables 2, 3 and 4), it is up to the clinician to assess what might be the most suitable software for the specific patient/protocol. We also have to emphasize that the presence of artifacts or relevant anatomical changes (bowel shape or filling, nasal cavity empty or full, etc.) could seriously affect the quality of the automatic contours generation.

Moreover, the three commercial software solutions have other differences that were not evaluated in this study. We can observe that A is the only one that doesn’t have its own contouring tools: another software to import and review the results of the deformation is needed. On the other hand A is a simple and straightforward software for automatic contour generation. Both V and M have tools for the deformable registration of CT images, cumulative dose volume histogram calculation, and V manages also the deformable registration of MRI with CT images (useful for treatment planning on brain, prostate and for paediatric patients), but we did not test the reliability of such tools. M required more time to learn how to operate the software.

In the context of on-line adaptive treatment, both automatic delineation of CTV and OARs are important. In general, we can say the higher the sensitivity of the OARs automatic segmentation, the lower the risk for over irradiation of the organs; the higher the sensitivity of the CTV segmentation, the lower the risk for under irradiation of tumor tissue. On the other hand, as discussed by Tsuji et al. [7], it is difficult to determine *a priori* whether automatic contours have acceptable accuracy because the importance lies also in the dosimetry of their resultant plans. Tsuji et al. found differences in target coverage and conformality with a similar range of DSC and also Voet et al. [6] found underdosages in the PTV of up to 11 Gy even for DSC coefficients of 0.8. Furthermore, in the Tsuji et al. statistical analysis, a significant correlation between the overlapping index, what we call “sensitivity index”, and the target coverage was shown. Tsuji et al. concluded that because of its stronger correlation with target coverage, the sensitivity index may be a better initial measure to predict contour utility, as opposed to DSC. We didn’t evaluate the dosimetric effects of our contour discrepancies, but we believe that each protocol for automatic recontouring should also be evaluated also from this point of view. This will be the goal for our future research.

As underlined in the literature [18], mapping planning contours to daily diagnostic CT images, instead of daily MVCT or kV CT, would facilitate adaptive replanning. Deformable image registration relies on image quality. In the present study, we used CT images from a fan-beam CT scanner. Our current automatic ROI delineation method can be directly applied to IGRT by CT-on-rail positioned in the treatment room. The image quality of Cone beam CT-CBCT is inferior to that of a fan-beam CT scanner. More importantly, the signal/noise ratio is dramatically low compared to the one of regular fan-beam CT images. If the contours are available on daily CT images, dose–volume histograms can be calculated to evaluate the necessity of replanning, or the contours can be used directly for intensity-modulated RT optimization. In addition, the daily dose distribution can be transformed back to the planning CT scan by using the same deformable image registration method to compare it to the original plan and estimate the cumulative doses delivered to the patient.

## Conclusion

The AC workflow was shown to be significantly shorter than the manual contouring process from scratch, even though manual correction of the VOIs is always needed. For the H&N site, a clinician can save about one hour, for a prostate patient, the time saved is about 40 minutes, and for a mesothelioma patient about 20 minutes. The differences, both in time and quality, between the software packages were statistically significant in many cases, but the absolute values of such differences are often modest.

## Competing interests

S. Gianolini is co-owner of MSS, the producer of VODCA.

## Authors’ contributions

MLM, FF, MA, and LW participated in study design. MLM, MA and MC made the manual contours. MLM, LW and FF drafted and wrote the manuscript. LW defined the equations and SG wrote the algorithm code for parameters evaluation. FF performed the automatic ROI propagation, collated the results and performed the statistical analysis. MA, MC, SG, VP and AJL reviewed/revised the manuscript. All authors read and approved the final manuscript.
